# Benign recurrent lymphocytic meningitis (Mollaret's meningitis) in Denmark: a nationwide cohort study

**DOI:** 10.1111/ene.16081

**Published:** 2023-10-05

**Authors:** Pelle Trier Petersen, Jacob Bodilsen, Micha Phill Grønholm Jepsen, Birgitte Rønde Hansen, Merete Storgaard, Lykke Larsen, Jannik Helweg‐Larsen, Lothar Wiese, Hans Rudolf Lüttichau, Christian Østergaard Andersen, Trine Hyrup Mogensen, Henrik Nielsen, Christian Thomas Brandt, for the Danish Study Group of Infections of the Brain (DASGIB)

**Affiliations:** ^1^ Department of Pulmonary and Infectious Diseases Nordsjællands Hospital Hillerød Denmark; ^2^ Faculty of Health and Medical Sciences University of Copenhagen Copenhagen Denmark; ^3^ Department of Infectious Diseases Aalborg University Hospital Aalborg Denmark; ^4^ Department of Clinical Medicine Aalborg University Aalborg Denmark; ^5^ Department of Infectious Diseases Hvidovre Hospital Hvidovre Denmark; ^6^ Department of Infectious Diseases Aarhus University Hospital Aarhus Denmark; ^7^ Department of Infectious Diseases Odense University Hospital Odense Denmark; ^8^ Department of Infectious Diseases, Rigshospitalet Copenhagen Denmark; ^9^ Department of Medicine Zealand University Hospital Roskilde Denmark; ^10^ Department of Infectious Diseases Herlev Hospital Herlev Denmark; ^11^ Department of Clinical Microbiology Hvidovre Hospital Hvidovre Denmark; ^12^ Department of Biomedicine Aarhus University Hospital Aarhus Denmark

**Keywords:** aciclovir, aseptic meningitis, herpes simplex virus type 2, viral meningitis

## Abstract

**Background and purpose:**

Data on clinical features and outcomes of benign recurrent lymphocytic meningitis (BRLM) are limited.

**Methods:**

This was a nationwide population‐based cohort study of all adults hospitalized for BRLM associated with herpes simplex virus type 2 (HSV‐2) at the departments of infectious diseases in Denmark from 2015 to 2020. Patients with single‐episode HSV‐2 meningitis were included for comparison.

**Results:**

Forty‐seven patients with BRLM (mean annual incidence 1.2/1,000,000 adults) and 118 with single‐episode HSV‐2 meningitis were included. The progression risk from HSV‐2 meningitis to BRLM was 22% (95% confidence interval [CI] 15%–30%). The proportion of patients with the triad of headache, neck stiffness and photophobia/hyperacusis was similar between BRLM and single‐episode HSV‐2 meningitis (16/43 [37%] vs. 46/103 [45%]; *p* = 0.41), whilst the median cerebrospinal fluid leukocyte count was lower in BRLM (221 cells vs. 398 cells; *p* = 0.02). Unfavourable functional outcomes (Glasgow Outcome Scale score of 1–4) were less frequent in BRLM at all post‐discharge follow‐up visits. During the study period, 10 (21%) patients with BRLM were hospitalized for an additional recurrence (annual rate 6%, 95% CI 3%–12%). The hazard ratio for an additional recurrence was 3.93 (95% CI 1.02–15.3) for patients with three or more previous episodes of meningitis.

**Conclusions:**

Clinical features of BRLM were similar to those of single‐episode HSV‐2 meningitis, whilst post‐discharge outcomes were more favourable. Patients with three or more previous episodes of meningitis had higher risk of an additional recurrence.

## INTRODUCTION

The attacks are relatively stereotyped. They begin suddenly with a violent headache (a feeling of a bar above the eyes); vomiting occurs, the temperature rises to 39°C, sometimes only to 38.5°C. The epileptic seizures have not ‘recurred’; however, on the first night, a slight delirium is possible, especially with hallucinations: figurative objects, people who are moving and working; perhaps all of this is only of relative interest as the patient has acquired the habit of asking his doctor for an injection of morphine since the first day of the attack. Nonetheless, improvement is evident the following day; the patient stays in bed for 48 h, and everything returns to normal. Some attacks are more attenuated and only last for a little more than half a day. Overall, the patient has become accustomed to his attacks; he speaks of them as an annoyance that he knows is inevitable but temporary. (Translated from reference 1)

In 1944, the French neurologist Pierre Mollaret described a previously unrecognized syndrome of recurrent episodes of meningitis with an unknown aetiology and a favourable outcome [1]. In addition to the benign and recurrent nature, Mollaret also noted that a type of large endothelial‐like cell was present in the cerebrospinal fluid (CSF) and thus named the syndrome benign multi‐recurrent endothelio‐leucocytic meningitis [1]. Later, herpes simplex virus type 2 (HSV‐2) was identified as the primary causative pathogen, and the syndrome is now referred to as Mollaret's meningitis or benign recurrent lymphocytic meningitis (BRLM) [2, 3, 4]. The endothelial‐like cells, which were found to be of monocytic origin, are called Mollaret's cells [5]. Although most patients with BRLM only experience a few episodes of meningitis, cases with more than 30 recurrences have been reported [5]. Patients are primarily younger or middle‐aged females, but factors influencing susceptibility on an individual level have only recently been explored [6, 7]. Furthermore, as clinical studies are limited to case reports and a few cohort studies of small patient populations, the clinical burden of BRLM remains unclear [2, 8, 9]. Thus, this nationwide cohort study described clinical features and outcomes of all adults hospitalized for BRLM associated with HSV‐2 at the departments of infectious diseases in Denmark from 2015 until 2020. Furthermore, BRLM was compared with single‐episode HSV‐2 meningitis.

## METHODS

### Design, setting and patients

The Danish Study Group of Infections of the Brain (DASGIB) database was used for this nationwide population‐based observational cohort study. Since 2015, all patients hospitalized for infections of the central nervous system at the departments of infectious diseases in Denmark have been prospectively included in the DASGIB database [10].

#### Benign recurrent lymphocytic meningitis

In the present study, all adult patients (≥18 years) hospitalized for BRLM associated with HSV‐2 between 1 January 2015 and 1 January 2020 were identified in the DASGIB database. The criteria for BRLM were adapted from a study by Kallio‐Laine et al. (regarding the number of episodes) and from the DASGIB database (regarding the characteristics of the episodes) [9, 10]. Patients were classified as having BRLM if they had experienced at least two episodes of HSV‐2 meningitis. Patients were also classified as having BRLM if they had experienced one episode of HSV‐2 meningitis in addition to one or more episodes of presumed but not microbiologically confirmed viral meningitis. HSV‐2 meningitis was defined as a clinical presentation of viral meningitis combined with either (i) detection of HSV‐2 DNA in the CSF by polymerase chain reaction (PCR), (ii) a CSF leukocyte count >10 cells/μL and positive intrathecal antibody index for HSV‐2 or (iii) a CSF leukocyte count >10 cells/μL and detection of HSV‐2 DNA from herpetic genital lesions by PCR. Presumed but not microbiologically confirmed viral meningitis was defined as a clinical presentation of viral meningitis combined with a CSF leukocyte count >10 cells/μL, and no other diagnosis was considered more likely given all available information. Since complete data on episodes of meningitis that occurred before the establishment of the DASGIB database were not available, categorization of aetiology was allowed to rely on the medical history registered in medical records. Patients were included with reference to the first recurrent episode of meningitis (i.e., the index episode) within the study period.

#### Single‐episode HSV‐2 meningitis

The comparison cohort consisted of all adult patients (≥18 years) in the DASGIB database hospitalized for HSV‐2 meningitis between 1 January 2015 and 1 January 2020 and with no history of previous episodes of viral meningitis. Patients with encephalitis were not included in either cohort [11].

### Variables

Data on clinical features, management and treatment were available in the DASGIB database. However, the number of previous episodes of meningitis experienced by each patient before inclusion was determined retrospectively by review of medical records. Alcohol abuse, intravenous substance abuse, organ transplantation, cancer (except skin cancer other than melanoma), diabetes mellitus, asplenia, human immunodeficiency virus infection, primary immunodeficiency, prednisolone >7.5 mg/day, or other immunosuppressive therapy constituted immunosuppression.

### Outcomes

Hospitalizations for additional recurrences of HSV‐2 meningitis or presumed viral meningitis were registered from the index episode (30 days after discharge) until 1 January 2021. Data on recurrences were obtained from medical records, which cover mortality and all public hospitalizations in Denmark via linkages to the Danish Civil Registration System and the Danish National Patient Register [12]. The Glasgow Outcome Scale (GOS) (1, death; 2, vegetative state; 3, severe disability; 4, moderate disability; 5, good recovery) was used to categorize functional outcomes which were available in the DASGIB database at discharge and at follow‐up visits approximately 30, 90 and 180 days after discharge. In the case of a missing GOS score, the last value was carried forward if patients had a GOS score of 5 at the most recent hospital contact. An unfavourable functional outcome was defined as a GOS score of 1–4.

### Statistical methods

Data were reported as median and interquartile range (IQR) or as count and percentage. The *χ* ^2^ test, Fisher's exact test, Kruskal−Wallis test or Mann–Whitney *U* test was used to compare data. Annual incidences of BRLM were calculated by dividing the number of patients with a first‐time recurrent episode of meningitis (i.e., incident cases) by the total adult population in Denmark in each year [13]. The progression risk from HSV‐2 meningitis to BRLM was estimated as the ratio between the number of incident cases of HSV‐2 meningitis and the number of incident cases of BRLM during the 5‐year inclusion period. The rate of hospitalizations for additional recurrences of meningitis amongst patients with BRLM was calculated by dividing the number of recurrences that occurred from the index episode until 1 January 2021 by the total person‐years. The Kaplan–Meier curve was used to visualize the timing of hospitalization for the first additional recurrence of meningitis amongst patients with BRLM. Cox proportional hazard models were used to estimate crude hazard ratios with 95% confidence intervals (CIs) of associations between potential risk factors and time (days) until hospitalization for the first additional recurrence after the index episode amongst patients with BRLM. Evaluated factors were age (18–30, ≥31), sex (male, female), the number of previous episodes of meningitis (2, ≥3), the triad of headache, stiff neck and photophobia and/or hyperacusis (yes, no), CSF leukocyte count (0–500, ≥501 cells/μL) and treatment with suppressive valacyclovir (yes, no). Patients were censored at loss to follow‐up or 31 December 2020, whichever came first. All *p* values were two‐sided, and significance levels were <0.05, except in comparisons of unfavourable functional outcomes where a Bonferroni‐adjusted significance level of 0.0125 was used. SAS Enterprise Guide 7.11 was used for all statistical analyses.

### Ethical approval

The DASGIB cohort was approved by the Danish Health Authority (record nos. 3‐3013‐2579/1 and 3‐3013‐3168/1). Consent from patients was not required.

## RESULTS

Of 54 patients with two or more episodes of viral meningitis identified in the DASGIB database, 47 patients fulfilled the study criteria for BRLM and were included for further assessment. In addition, 125 patients with HSV‐2 meningitis and no history of previous episodes of viral meningitis were identified in the DASGIB database. Seven of these patients had a recurrent episode of meningitis during the study period and were included in the BRLM cohort, thus leaving 118 patients with single‐episode HSV‐2 meningitis as a comparison cohort.

At the time of inclusion, and counting the index episode, 28 (60%) of 47 patients with BRLM had two previous episodes of meningitis, six (13%) patients had three and 13 (28%) patients had more than three. Amongst the 47 patients with BRLM, HSV‐2 was detected during the index episode in 41 (87%) and solely during a previous episode of meningitis in the remaining six (13%). During the index episode, PCR for herpes simplex virus type 1 and 2, varicella‐zoster virus and enteroviruses on CSF was done in all patients with BRLM. Based on the 28 incident cases of BRLM, the mean annual incidence was 1.2 cases per 1,000,000 adults. The estimated progression risk from HSV‐2 meningitis to BRLM was 22% (95% CI 15%–30%).

**FIGURE 1:**
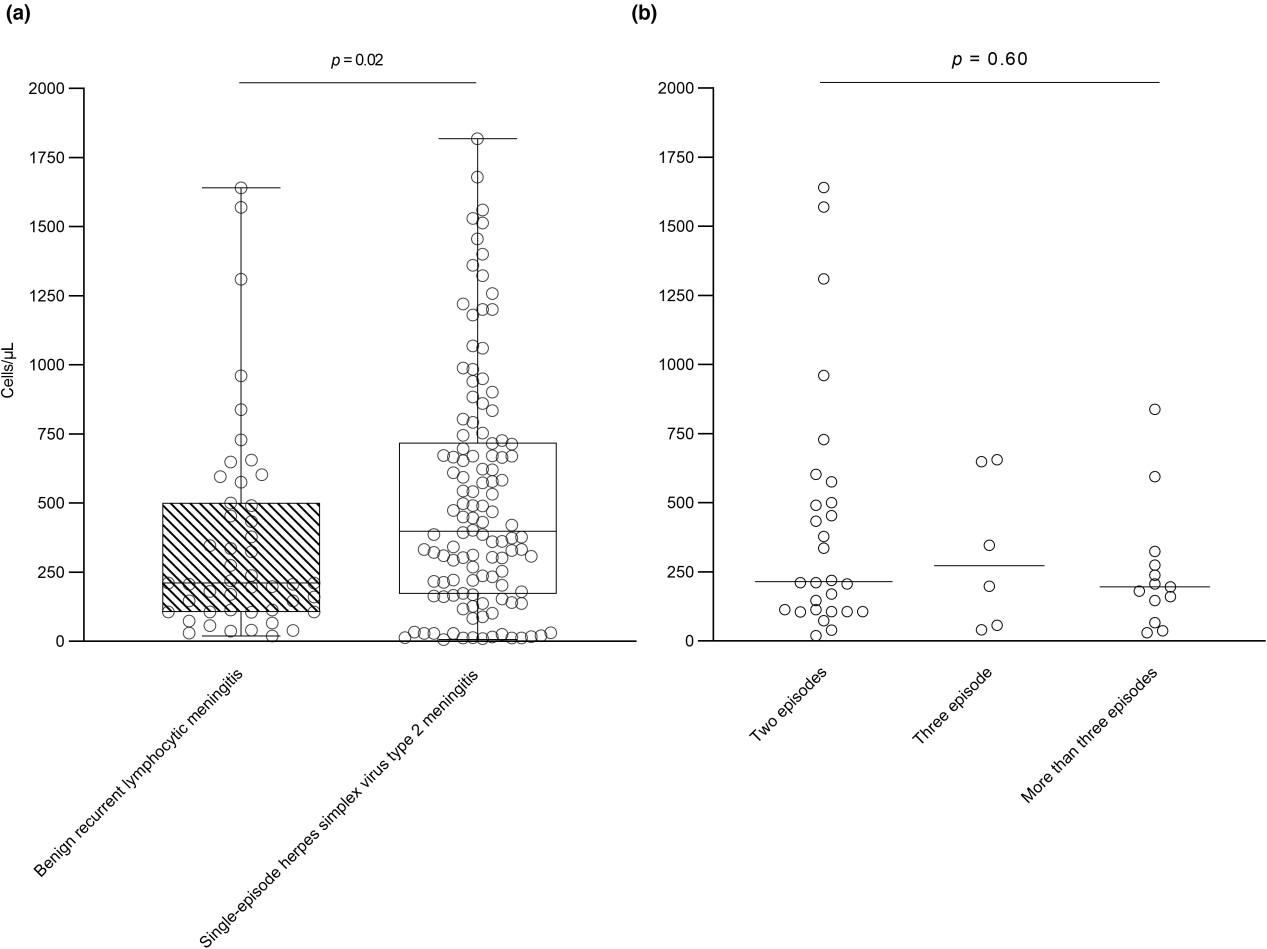
Cerebrospinal fluid leukocyte counts in adults with benign recurrent lymphocytic meningitis and single‐episode herpes simplex virus type 2 meningitis (a) and by the number of previous episodes of meningitis (b).

The median age was higher in patients with BRLM than in those with single‐episode HSV‐2 meningitis (44 years [IQR 31–50] vs. 34 years [IQR 26–48]; *p* = 0.04), whereas the proportions of females and patients with immunosuppression were similar (Table 1). Patients with BRLM had a shorter median duration of symptoms before admission than those with single‐episode HSV‐2 meningitis (1 day [IQR 1–2] vs. 2 days [IQR 1–4]; *p* = 0.03). The proportion of patients with the triad of headache, stiff neck and photophobia and/or hyperacusis was similar between BRLM and single‐episode HSV‐2 meningitis (16/43 [37%] vs. 46/103 [45%]; *p* = 0.41). Patients with BRLM had a lower median CSF leukocyte count than those with single‐episode HSV‐2 meningitis (221 cells [IQR 107–500] vs. 398 cells [IQR 174–716]; *p* = 0.02), but the CSF leukocyte count was otherwise not associated with the number of previous episodes of meningitis amongst patients with BRLM (Figure 1). Amongst the 47 patients with BRLM, two (4%) had a neutrophil percentage >50%, both with microbiologically confirmed HSV‐2 meningitis.

**TABLE 1:** Clinical features on admission in adults with benign recurrent lymphocytic meningitis and single‐episode herpes simplex virus type 2 meningitis.

Clinical features	Benign recurrent lymphocytic meningitis	Single‐episode herpes simplex virus type 2 meningitis	*p* value
	*N* = 47	*N* = 118	
Age, years	44 (31–50)	34 (26–48)	0.04
Sex, female	40/47 (85)	87/118 (74)	0.12
Full‐time occupation	32/45 (71)	90/112 (80)	0.21
Immunosuppression	5/47 (11)	8/118 (7)	0.52
Duration of symptoms before admission, days	1 (1–2)	2 (1–4)	0.03
Headache	46/47 (98)	109/117 (93)	0.45
Neck stiffness	26/45 (58)	61/111 (55)	0.75
Photophobia/hyperacusis	32/45 (71)	80/107 (75)	0.64
History of fever	23/43 (53)	83/104 (80)	0.001
Temperature ≥38°C	18/47 (38)	53/114 (46)	0.34
GCS score <15^a^	1/46 (2)	7/116 (6)	0.44
B‐leukocytes, cells ×10^9^/L	7.8 (6.6–9.1)	8.6 (7.0–10.6)	0.19
C‐reactive protein >10 mg/L	3/43 (7)	20/113 (18)	0.09
CSF leucocytes, cells/μL	211 (107–500)	398 (174–716)	0.02
CSF neutrophil percentage	3 (1–14)	3 (1–8)	0.64
CSF protein, g/L	0.86 (0.70–1.50)	1.19 (0.80–1.50)	0.15

*Note*: Quantitative data are median (interquartile range) and categorical data are *n*/*N* (%).

Abbreviations: B, blood; CSF, cerebrospinal fluid.

^a^ Glasgow Outcome Scale score <15 for <24 h.

The median time from admission until lumbar puncture was shorter for patients with BRLM than for those with single‐episode HSV‐2 meningitis (1.3 h [IQR 0.8–3.1] vs. 2.6 h [IQR 1.2–5.6]; *p* = 0.009). Most patients were treated with intravenous aciclovir or valaciclovir during admission (46/47 [98%] with BRLM vs. 113/118 [96%] with single‐episode HSV‐2 meningitis; *p* = 0.68), but the median time from admission until treatment was shorter for patients with BRLM than for those with single‐episode HSV‐2 meningitis (3 h [IQR 2–8] vs. 7 h [IQR 3–22]; *p* = 0.003). Suppressive valaciclovir was not used by any patient with BRLM at the time of inclusion but was initiated in the immediate extension of the index episode in 11 (25%) of 44. Cranial imaging was done in 11 (23%) of 47 patients with BRLM, and except for an arachnoid cyst in a patient with PCR‐proven HSV‐2 meningitis, no abnormalities were observed. The median length of hospitalization was 3 days (IQR 1–5) for patients with BRLM and 4 days (IQR 2–7) for those with single‐episode HSV‐2 meningitis (*p* = 0.07).

**FIGURE 2:**
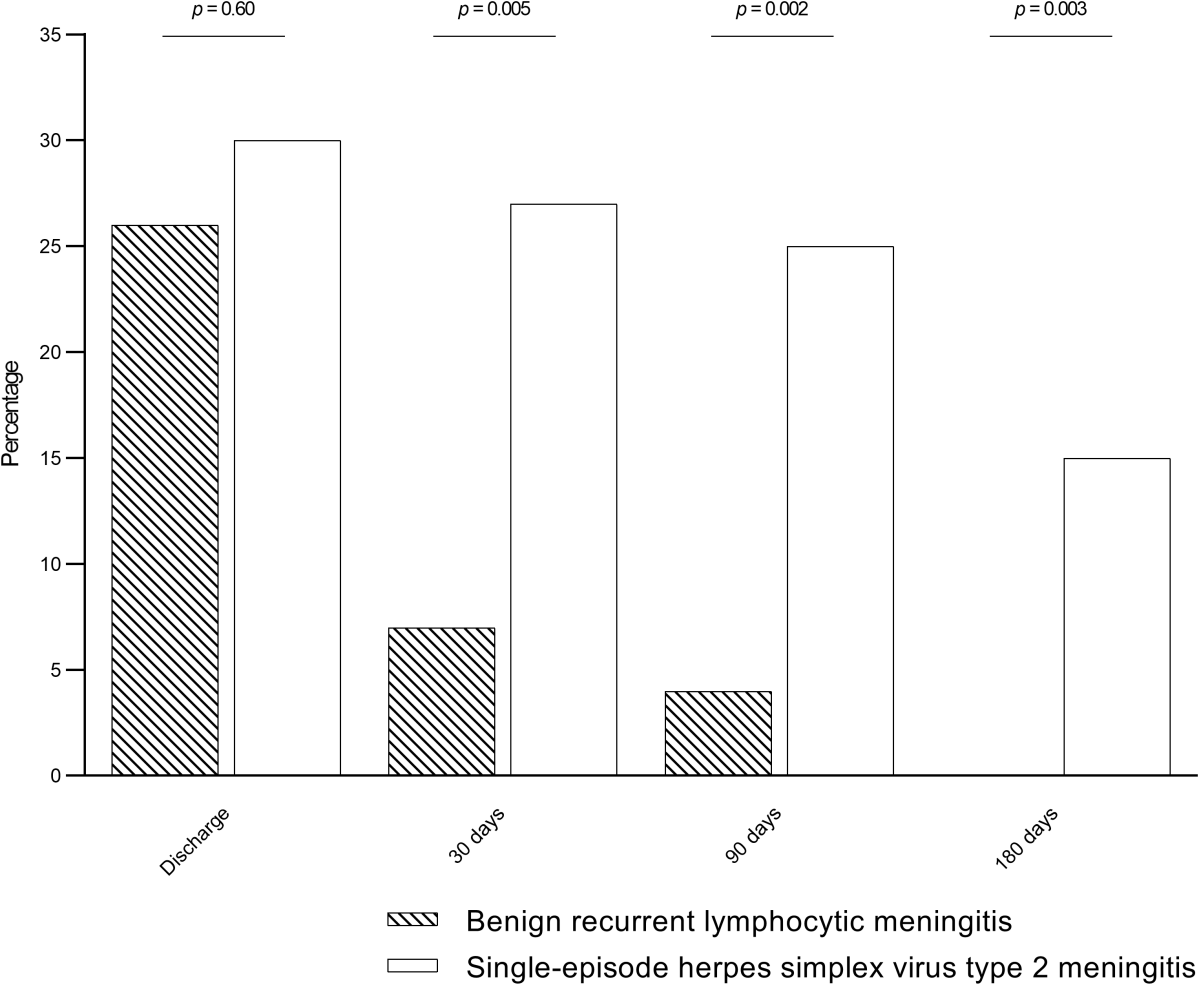
Unfavourable functional outcomes (Glasgow Outcome Scale scores of 1–4) by time after discharge in adults with benign recurrent lymphocytic meningitis and single‐episode herpes simplex virus type 2 meningitis.

No patient with BRLM had a GOS score <4 at discharge or at any follow‐up visit. Whilst the proportion of patients with an unfavourable functional outcome (GOS score of 1–4) was similar between BRLM and single‐episode HSV‐2 meningitis at discharge (12 [26%] of 47 vs. 35 [30%] of 118; *p* = 0.60), it was less frequent in BRLM at 30 days (3 [7%] of 45 vs. 31 [27%] of 116; *p* = 0.005), 90 days (2 [4%] of 46 vs. 29 [25%] of 114; *p* = 0.002) and 180 days after discharge (0 [0%] of 46 vs. 16 [15%] of 104; *p* = 0.003; Figure 2). These differences were overall consistent when the comparisons were restricted to patients with premorbid full‐time occupations, although only statistically significant at 90 days after discharge (Figure S1).

**FIGURE 3:**
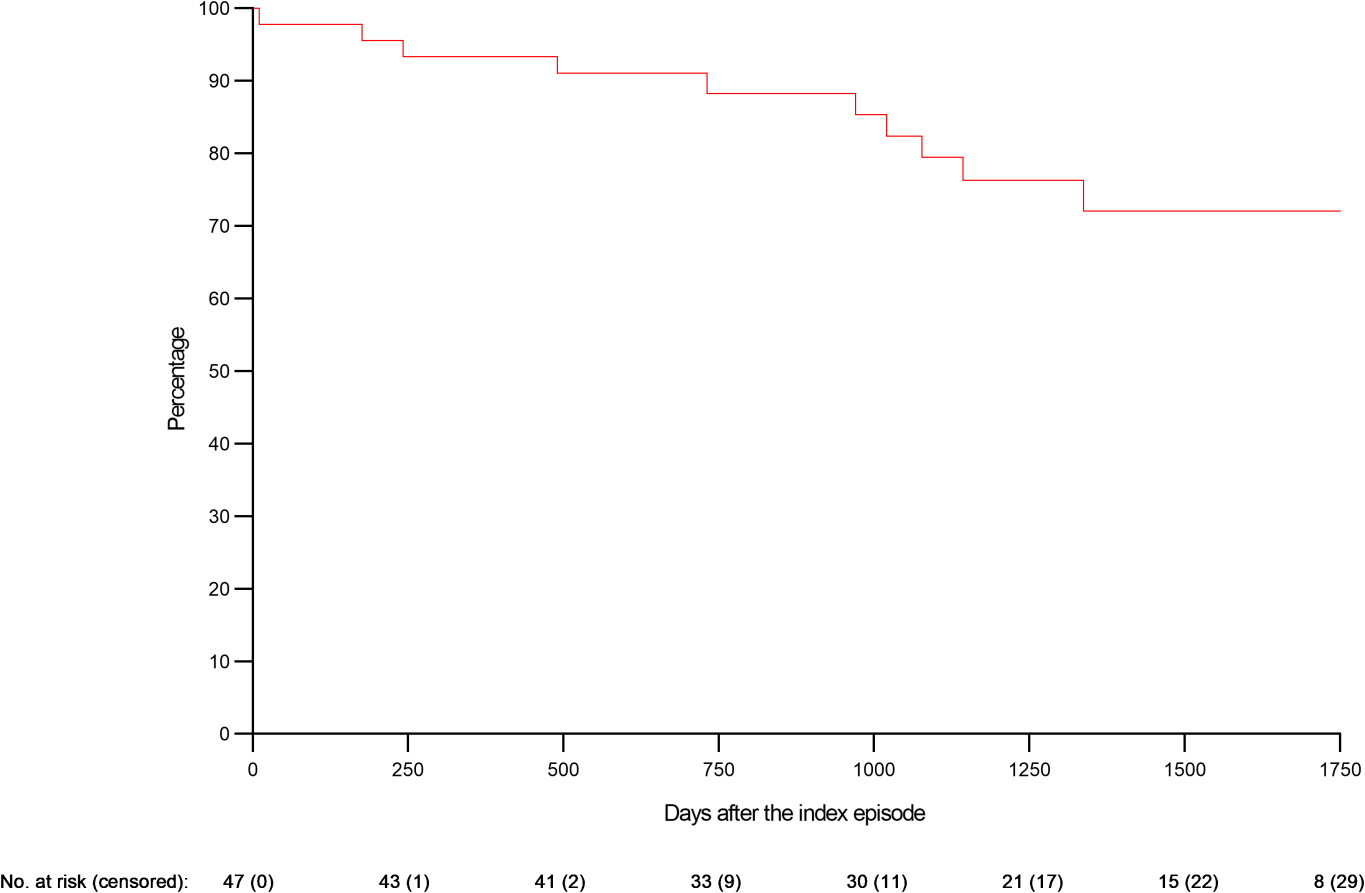
Kaplan–Meier curve for additional recurrences of meningitis in adults with benign recurrent lymphocytic meningitis.

During a median follow‐up of 1302 days (range 0–2052 days), 10 (21%) of 47 patients with BRLM were hospitalized for an additional recurrence of meningitis, which yielded an annual rate of 6% (95% CI 3%–12%). No patients were hospitalized more than once for additional recurrences during the study period. The timing of additional recurrences is shown with a Kaplan–Meier curve (Figure 3). Using patients with two previous episodes of meningitis as the reference, the hazard ratio of having an additional recurrence was 3.93 (95% CI 1.02–15.3) for patients with three or more previous episodes (Table 2). No other evaluated factor was associated with the risk of an additional recurrence.

**TABLE 2:** Risk factors for first additional recurrence of meningitis amongst adults with benign recurrent lymphocytic meningitis.

Risk factor	Hazard ratio for additional recurrence (95% confidence interval)
Age, years	
18–30	Reference
≥31	0.91 (0.19–4.32)
Sex	
Male	Reference
Female	0.33 (0.09–1.29)
Number of previous episodes	
2	Reference
≥3	3.93 (1.02–15.3)
Triad of signs and symptoms^a^	
No	Reference
Yes	1.68 (0.49–5.81)
CSF leukocyte count, cells/μL	
0–500	Reference
≥501	0.82 (0.18–3.89)
Suppressive valacyclovir	
No	Reference
Yes	1.06 (0.27–4.14)

*Note*: Quantitative data are median (interquartile range) and categorical data are *n*/*N* (%).

Abbreviation: CSF, cerebrospinal fluid.

^a^ Triad of headache, neck stiffness, and photophobia and/or hyperacusis.

## DISCUSSION

In this nationwide cohort study, 47 patients hospitalized for BRLM associated with HSV‐2 were included during a 5‐year period. The clinical presentation of BRLM was similar to single‐episode HSV‐2 meningitis, but post‐discharge functional outcomes were more favourable. In BRLM, the risk of an additional recurrence of meningitis was higher for patients with three or more previous episodes of meningitis than for those with only two.

The definition of BRLM varies amongst studies. Unlike some previous studies, a predominant lymphocytic CSF cell count was not used as a criterion for BRLM in this study, and a minimum of only two episodes of meningitis (instead of three) were required [2]. Additionally, the present study included only patients where BRLM was associated with HSV‐2 and based on 28 incident cases; the annual incidence was 1.2 cases per 1,000,000 adults. The incidence of BRLM has not been previously reported, but a study from Finland found an 11‐year prevalence (i.e., not only incident cases) of HSV‐2 associated BRLM of 2.2 cases per 100,000 persons [9]. In the present study, the progression risk from HSV‐2 meningitis to BRLM was estimated at 22%, which was similar to results from a study from Sweden, where 19% of patients with HSV‐2 meningitis without a history of previous episodes of meningitis experienced a recurrence [8]. A few other studies have also estimated the risk of recurrences of meningitis (from 20% to 32%); however, these results were not only based on patients with a first‐time episode of HSV‐2 meningitis but also included cases with BRLM [9, 14, 15].

Consistent with previous studies, the CSF leukocyte count was lower in BRLM than in single‐episode HSV‐2 meningitis in this study [8, 16]. However, the less pronounced CSF inflammation was not mirrored in the clinical presentation, which was similar to single‐episode HSV‐2 meningitis except that fever before admission was less common. Both a lower CSF leukocyte count and a lower prevalence of fever could result from the shorter duration of symptoms before admission experienced by patients with BRLM. Yet, a tendency towards a shorter course of hospitalization was observed for BRLM, suggesting that recurrent episodes of meningitis resolve faster than single episodes. This is equivalent to the natural history of genital HSV‐2 infection, where the duration of symptoms decreases with recurrent attacks [17]. The patients' familiarity with the disease, along with a timely diagnosis, could also have reduced both the time before admission and the length of hospitalization in BRLM.

In this study, functional outcomes were less frequently unfavourable for patients with BRLM than for those with single‐episode HSV‐2 meningitis at all outpatient follow‐up visits, and almost all patients with BRLM had returned to normal daily living (i.e., a GOS score of 5) within 30 days of discharge. This could indicate that sequelae related to BRLM are, by nature, less severe than those of single‐episode HSV‐2 meningitis. Alternatively, the shorter time until antivirals could have improved the functional outcome in BRLM. However, no association between early antiviral treatment and improved outcomes in meningitis caused by HSV‐2 or varicella‐zoster virus was observed in a recent study [18].

Whilst no previous study has evaluated potential risk factors for additional recurrences in BRLM, younger age, male sex and the severity of the initial infection have been associated with an increased risk of new attacks in genital HSV‐2 infection [19]. In this study, having three or more previous episodes of meningitis was a risk factor for an additional episode, whilst neither age, sex, the burden of signs and symptoms (i.e., the triad of headache, neck stiffness and photophobia and/or hyperacusis), nor the degree of the subarachnoid inflammation (i.e., the CSF leukocyte count) were associated with new recurrences. Furthermore, initiation of suppressive aciclovir was not associated with a lower risk of recurrence. In line with this observation, suppressive valaciclovir had no beneficial effect on recurrences when evaluated in a randomized controlled trial in patients with HSV‐2 meningitis [14].

### Limitations

This study had limitations. First, the incidence of BRLM may have been underestimated since only patients admitted to departments of infectious diseases were included. Secondly, this study did not include patients diagnosed with BRLM based on the presence of Mollaret's cells in the CSF, although this has previously been reported [20]. Thirdly, information on previous episodes of meningitis that occurred before the establishment of the DASGIB database was retrospective in nature and thus at risk of misclassification. Fourthly, the GOS was not developed to evaluate sequelae after viral meningitis and might be too crude to capture the more subtle neurocognitive impairments observed amongst these patients [21, 22]. Fifthly, as the GOS assesses a decline in functional capacity from a premorbid level, the observed differences between BRLM and single‐episode HSV‐2 meningitis could reflect that patients with BRLM have adjusted daily living to the disease, thus not experiencing any further decline after each additional episode of meningitis. However, the differences in functional outcomes were overall consistent when only patients with premorbid full‐time occupations were compared. Furthermore, some patients with BRLM might defer hospital admission and instead await recovery at home. This would underestimate both incidence and recovery in BRLM and thus supports that these patients have an improved functional outcome compared with those with single‐episode HSV‐2 meningitis. Finally, in analyses of risk factors for an additional recurrence, data on adherence to suppressive antiviral treatment after initiation were unavailable.

## CONCLUSIONS

Benign recurrent lymphocytic meningitis associated with HSV‐2 is a rare disease, but almost one in four patients with a first episode of HSV‐2 meningitis will at some point experience a recurrence. Whilst most clinical features of BRLM were similar to those of single‐episode HSV‐2 meningitis, the functional outcome was more favourable. Only the number of previous episodes of meningitis was associated with the risk of an additional recurrence amongst patients with BRLM.

## AUTHOR CONTRIBUTIONS

Pelle Trier Petersen: Writing—original draft; conceptualization; methodology; investigation; funding acquisition; formal analysis. Jacob Bodilsen: Conceptualization; methodology; investigation; supervision; writing—review and editing. Micha Phill Grønholm Jepsen: Conceptualization; investigation; methodology; supervision; writing—review and editing. Birgitte Rønde Hansen: Investigation; writing—review and editing. Merete Storgaard: Investigation; writing—review and editing. Lykke Larsen: Investigation; writing—review and editing. Jannik Helweg‐Larsen: Investigation; writing—review and editing. Lothar Wiese: Investigation; writing—review and editing. Hans Rudolf Lüttichau: Investigation; writing—review and editing. Christian Østergaard Andersen: Investigation; writing—review and editing. Trine Hyrup Mogensen: Investigation; writing—review and editing. Henrik Nielsen: Investigation; writing—review and editing. Christian Thomas Brandt: Conceptualization; investigation; methodology; writing—review and editing; supervision; funding acquisition.

## FUNDING INFORMATION

This work was supported by Helsefonden (grant number 21‐B‐0437), Helen Rudes Fond (grant number 60988), A & J C Tvergaards Fond, Minister Erna Hamiltons Legat for Videnskab og Kunst (grant number 24‐2022) and Nordsjællands Hospital to PTP.

## CONFLICT OF INTEREST STATEMENT

The authors declare no conflicts of interest.

## Supporting information


Figure S1.

## Data Availability

Data are only available with permission from the Danish Health Authority.
