# Supplementary material for: Benign recurrent lymphocytic meningitis (Mollaret's meningitis) in Denmark: a nationwide cohort study
Source: Eur J Neurol. 2023 Oct 5;31(1):e16081. doi: 10.1111/ene.16081 (PMC11235955; doi:10.1111/ene.16081)

Supplementary material to: Benign recurrent lymphocytic meningitis (Mollaret's meningitis) in Denmark: a nationwide cohort study

Supplementary Figure 1. Unfavourable functional outcomes (Glasgow Outcome Scale scores of 1-4) by time after discharge in adults with benign recurrent lymphocytic meningitis and single-episode herpes simplex virus type 2 (only premorbid full-time occupations).

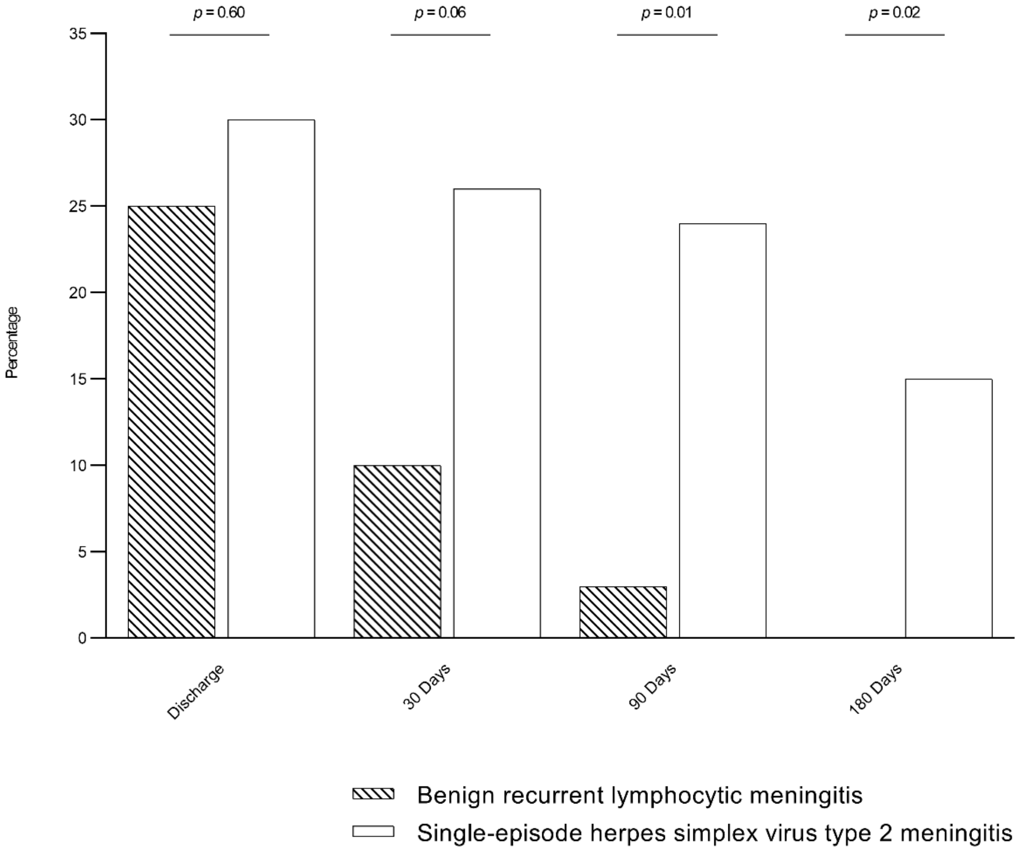

Supplement: Supplementary file 1 — Figure S1. [file ENE-31-e16081-s001.pdf]
